# Late-onset hypogonadism and lower limb vascular complications in men with type 2 diabetes: an observational study in a tertiary care centre

**DOI:** 10.1007/s40618-026-02967-4

**Published:** 2026-06-26

**Authors:** Rita Matrone, Paola Caruso, Miriam Longo, Alessandro Pontillo, Carla Carbone, Paolo Cirillo, Chiara Porcellini, Maria Tomasuolo, Lorenzo Scappaticcio, Maria Ida Maiorino, Giuseppe Bellastella, Katherine Esposito

**Affiliations:** 1 Department of Advanced Medical and Surgical Sciences, University of Campania “Luigi Vanvitelli”, Naples, Italy; 2 Department of Experimental Medicine, University of Campania “Luigi Vanvitelli”, Naples, Italy; 3 Division of Endocrinology and Metabolic Diseases, University of Campania “Luigi Vanvitelli”, Piazza Luigi Miraglia 2, 80138 Naples, Italy

**Keywords:** Late-onset hypogonadism, Lower-limb complications, Type 2 diabetes, Peripheral artery disease, Diabetic foot ulcer

## Abstract

**Purpose:**

The aim of this study was to evaluate the relationship between late onset hypogonadism (LOH) with inflammation and lower-limb vascular complications in men with type 2 diabetes (T2D).

**Methods:**

This single-centre, retrospective observational study included men with T2D who underwent hormonal evaluation between 2020 and 2024. LOH was diagnosed according to SIAMS/SIE and EAU guidelines and participants were classified as hypogonadal or eugonadal. Demographic, metabolic, inflammatory, and haematological parameters were collected. Lower-limb vascular complications, including peripheral artery disease (PAD), diabetic foot ulcer (DFU), osteomyelitis and amputation, were assessed. Logistic regression analyses were performed to evaluate the association between testosterone (T) levels and vascular complications.

**Results:**

The study population included 155 men, with a prevalence of LOH of 50.3%. Hypogonadal men showed significantly lower haemoglobin and haematocrit levels and higher inflammatory markers compared with eugonadal subjects. Microvascular complications did not differ between groups, except for a higher prevalence of DFU in hypogonadal men. Conversely, macrovascular lower-limb complications were more frequent in men with LOH, including PAD, osteomyelitis, and amputation. Higher T levels were associated with a reduced number of these outcomes. After multivariable adjustment, T levels remained independently associated with the risk of PAD and lower limb amputation.

**Conclusion:**

In our cohort of men with T2D, hypogonadism is associated with an increased burden of macrovascular and lower limb complications. Lower T levels were independently associated with PAD and amputation, supporting hypogonadism as a marker of vascular impairment.

**Supplementary Information:**

The online version contains supplementary material available at https://doi.org/10.1007/s40618-026-02967-4.

## Introduction

Male hypogonadism is a relatively common condition resulting from insufficient testosterone (T) production and/or impaired spermatogenesis due to primary testicular disorders or dysfunction of the hypothalamic–pituitary–gonadal (HPG) axis [1]. Clinical manifestations depend on the timing and severity of androgen deficiency and, in post-pubertal cases, commonly include sexual dysfunction, reduced fertility, fatigue, adverse changes in body composition and bone mass [1, 2]. Its prevalence has increased substantially in recent decades, particularly in adult and aging male populations [3].

In 2004, Dhindsa et al. described a frequent association between hypogonadotropic hypogonadism and type 2 diabetes (T2D) in males [4]. Typically emerging in later life, this condition is characterized by decreased T levels along with inappropriately low Follicle-Stimulating Hormone (FSH) and Luteinizing Hormone (LH) secretion, despite a history of normal pubertal development and secondary sexual traits [5, 6]. This status, known as Late-Onset Hypogonadism (LOH), appears to affect a substantial subset of men with T2D, with an estimated prevalence ranging from 25 to 40% [7].

Moreover, potential pathophysiological mechanisms underlying the association between the HPG axis and metabolic syndrome have been described: excess adiposity, with regard to visceral fat, plays a pivotal role in this endocrine-metabolic disruption. Specifically, the interplay of adipose tissue with leptin and insulin resistance promotes a chronic low-grade inflammatory state, which may negatively impact the HPG axis. On the other hand, serum levels of total T have been inversely correlated with the number of components of metabolic syndrome [8, 9]. Particularly, insulin resistance may be considered one of the most relevant causative factors in obesity-induced hypogonadism. It is known from studies on cell cultures that insulin stimulates the HPG axis activity at several levels, i.e., hypothalamic, pituitary and testicular levels [5, 8]. Moreover, insulin may also influence hypothalamic neurons, with consequent effects on the gonadotropin-releasing hormone (GnRH) secretion [10]. However, LOH is also present in 25% of non-obese diabetic males, suggesting that the finding of low T concentration is not entirely dependent on obesity [7]. In this context, a primary dysfunction at the hypothalamo–hypophyseal level has been hypothesized. Notably, a high prevalence of circulating anti-pituitary antibodies has been reported in men with T2D, particularly among those with hypogonadotropic hypogonadism, supporting a potential autoimmune-mediated impairment of pituitary gonadotroph cells and pointing to pituitary autoimmunity as an additional pathogenic mechanism [11].

Beyond central autoimmunity, chronic hyperglycaemia itself may independently contribute to the reduction in T levels, together with other risk factors including age > 60 years, obesity, hypertriglyceridemia, reduced glomerular filtration rate (< 60 ml/min/1.73m^2^) and abnormal liver function tests [12–14]. Moreover, hyperglycaemia has been described as a possible determinant of disrupted fertility, based on impaired function of the HPG-axis, increased DNA-damage, perturbations in the advanced glycation end-products system, high oxidative and endoplasmic reticulum stress, modulation of cellular pathways, impaired mitochondrial function and disrupted sympathetic innervation [15].

Among diabetes-related vascular complications, those involving the lower limbs represent a particularly severe and disabling clinical burden, being closely associated with ulceration, infection, and amputation. Although the relationship between T2D and hypogonadism has been so far investigated, evidence about the effect of LOH on lower-limb vascular complications of diabetes is limited. A reduction in T levels has been frequently observed in people with T2D and microvascular complications, including diabetic neuropathy and retinopathy [16, 17]. However, previous studies reported the connection between hypogonadism and macrovascular complications in men with T2D [12, 18–21].

Furthermore, a recent review regarding cardiovascular risk in men with hypogonadism reported significant health benefits provided by T therapy, with a reduction in cardiovascular risk according to the duration of therapy [22].

The aim of this study was to evaluate the potential relationship between T levels, inflammation markers and lower-limb vascular complications in a population of males with T2D.

## Materials and methods

This single-centre, retrospective, observational study was conducted at the Endocrinology and Metabolic Diseases Unit at the University Hospital of Campania “Luigi Vanvitelli” (Naples, Italy), collecting data on males with a diagnosis of T2D according to American Diabetes Association guidelines [23] who performed hormonal evaluation with regard to gonadal function from 2020 to 2024. The study was approved by the Medical Ethics Committee of University of Campania. We excluded people affected by acute systemic illness, severe chronic kidney disease or liver disease, other forms of diabetes and/or new-onset diabetes, people with other forms of hypogonadism (i.e. secondary to pituitary disease or in androgen-deprivation therapy), or on testosterone replacement therapy. The study complies with the Declaration of Helsinki and the International Conference on Harmonization Good Clinical Practice guidelines. In accordance with the regulations followed by the ethics committee, all study participants had to provide signed informed consent.

### Data collection

Data were collected retrospectively from medical records and organized into an internal electronic database. Age, diabetes duration, smoking habit and pharmacological therapies of all patients were recorded. Anthropometric parameters and information about glycaemic control [fasting plasma glucose and glycated haemoglobin (HbA1c)], renal function [serum creatinine, estimated glomerular filtration rate (eGFR) and urinary albumin-to-creatinine ratio (UACR)], lipid profile (total cholesterol, triglycerides, HDL-cholesterol, LDL-cholesterol), blood count, coagulation (PT, aPTT, INR, fibrinogen), inflammatory markers (ESR, CRP, fibrinogen) were gathered. Moreover, data about gonadal function, including total T, SHBG, LH and FSH levels were collected in the fasting state, in the morning between 08.00 am and 11.00 am, to avoid interference with daily hormonal variability. All the assays were performed in the hospital’s chemistry laboratory.

The presence of long-term micro- and macrovascular complications was evaluated according to standard medical practice. Diagnosis of diabetic retinopathy was performed by the fundus oculi exam in mydriasis with a slit-lamp. Diabetic kidney disease (DKD) was diagnosed by the persistent presence of elevated UACR (≥ 30 mg/g Cr) and/or low eGFR (< 60 ml/min/1.73 m^2^), without concomitant signs of other forms of renal disease. Diabetic neuropathy was evaluated with the diabetic neuropathy index for somatic and autonomic neuropathy. Cardiovascular disease was evaluated through medical history, electrocardiogram and expert advice with echocardiogram. Data on cerebrovascular disease were collected from the medical history and secondary to specialist advice when indicated. The presence of peripheral artery disease (PAD) was assessed through peripheral Doppler ultrasound, transcutaneous oximetry of the lower limbs, along with the examination and palpation of peripheral pulses. In people with diabetic foot ulceration (DFU), information about ulcer duration, presence of infection/osteomyelitis and requirement for surgical interventions was collected. Data on LOH-related symptoms, including decreased sexual desire, erectile dysfunction, reduced frequency of spontaneous erections, reduced or absent ejaculation, fatigue and other related manifestations, were derived from clinical evaluations documented in the medical records.

### Diagnosis of LOH

The study population was divided into groups depending on the diagnosis of LOH or eugonadal status, according to the clinical practice guidelines of the Italian Society of Andrology and Sexual Medicine (SIAMS) and the Italian Society of Endocrinology (SIE) for hypogonadotropic hypogonadism and the European Association of Urology (EAU) [24, 25]. LOH was diagnosed in men with normal puberty development, specific symptoms as listed above, and at least two measurements of morning total T < 230 ng/dl. Men with T levels > 350 ng/dl were considered eugonadal. In males with a total T ranging between 230 ng/dl and 350 ng/dl, the Vermeulen formula [26] was performed to calculate free T (fT), considering as threshold fT levels < 220 pmol/L to define fT deficiency [27]. Hypogonadal individuals underwent LH evaluation, in order to exclude primary hypogonadism and confirm the diagnosis of LOH (LH values < 9.4 IU/L).

### Statistical analysis

Descriptive statistics were used to characterize the study population and to compare baseline parameters between individuals with hypogonadism and those who were eugonadal. The Kolmogorov–Smirnov test was used to assess the variables distribution. Data in tables and figures were reported as mean ± standard deviation (SD) or median and interquartile range (IQR), depending on the distribution of the sample. Differences at the baseline between groups were evaluated by two-sided Student’s *t* test or Wilcoxon–Mann–Whitney test, as appropriate. The χ^2^ test was used to compare dichotomous variables. Binary logistic regression analysis was performed to define the association of T levels with the macrovascular complications which resulted statistically significant in the comparative analysis between the groups. Subsequently, a multivariable regression analysis adjusted for the potential confounding factors was carried out by building models in which variables were inserted progressively. Confounders were selected based on significant differences between groups and on their potential to influence both testosterone levels and vascular outcomes. These included HDL cholesterol, haemoglobin, haematocrit, platelet count, age, smoking status, body mass index (BMI), duration of diabetes, HbA1c, renal function (serum creatinine, UACR, and eGFR), and the use of cardiovascular medications, including angiotensin receptor blockers (ARBs) and β-blockers. The relationship between gonadal status and complications of diabetes was evaluated using the Odds Ratio (ORs). To improve clinical interpretability of the results, ORs for T were expressed per 50 ng/dL increase, obtained by exponentiating the original logistic regression coefficient. A post-hoc analysis was performed to estimate the ability of the study to detect differences in micro- and macrovascular complications of diabetes between groups. Considering an α-level of 0.05 and effect sizes comparable to those reported in a previous study [20], the available sample size was deemed sufficient to ensure an adequate statistical power. The statistical significance was recognized for values of *p* < 0.05. All analyses were conducted with SPSS software (version 25.0, SPSS, Chicago, IL, USA).

## Results

The study population included 155 men, with a median age of 61 years (IQR 53–67) and a median duration of diabetes of 14 years (IQR 7–20). In this cohort, the prevalence of LOH was 50.3%, resulting in an approximately equal division of the population. Patients were divided into 2 groups according to gonadal status [group E (eugonadal) = 77 individuals and group H (hypogonadal) = 78]. Baseline parameters are reported in Table 1. Groups did not differ in age, duration of diabetes, glycaemic control, kidney function and body mass index. The proportion of smokers was significantly higher in the E group (*p* = 0.036), as compared with the H group. Moreover, participants in the H group presented significantly lower levels of haemoglobin (*p* < 0.001) and haematocrit (*p* = 0.003), and higher levels of inflammation markers, including white blood cells (*p* < 0.001), C-reactive protein (CRP) (*p* < 0.001), fibrinogen (*p* < 0.001), erythrocyte sedimentation rate (ESR) (*p* < 0.001) and platelets (*p* = 0.042), as compared with men in the E group. Blood lipids levels did not present significant differences between groups, except for HDL cholesterol levels (*p* = 0.001) which were higher in the E group. Furthermore, there were no significant differences in administration of pharmacological therapies, with the exception of ARBs (*p* = 0.012) and β-blockers (*p* = 0.012), both significantly more frequently used in the H group.

**Table 1:** Clinical characteristics of the study participants

Parameters	Hypogonadal (78)	Eugonadal (77)	*p*-value
T, ng/dl	188 (125.0, 225.0)	454 (405.3, 541.8)	**< 0.001**
Age, years	61.0 (54.0, 67.0)	61.0 (52.7, 68.0)	0.947
Smoking, n (%)	55 (70.5)	66 (85.7)	**0.036**
Duration of diabetes, years	15 (7, 20)	12 (7, 19)	0.329
Weight, kg	86.75 (78.0, 100.9)	82.5 (73.6, 98.6)	0.063
BMI, kg/m^2^	29.7 (27.3, 34.3)	28.1 (25.1, 31.9)	0.100
SBP, mmHg	130 (120, 140)	130 (120, 140)	0.396
DBP, mmHg	72.5 (70, 80)	80 (70, 85)	0.724
Heart rate, bpm	77 (70.0, 85.3)	75 (68.0, 80.7)	0.148
Glycaemic control			
HbA1c, %	8.4 (7.1, 9.72)	8.2 (6.8, 9.5)	0.328
Fasting glucose, mg/dL	159.5 (113.0, 205.0)	139.5 (109.5, 207.5)	0.383
Lipid profile			
Total cholesterol, mg/dL	153.8 ± 41.6	165.4 ± 41.3	0.274
LDL cholesterol, mg/dL	87.3 ± 35.3	94.9 ± 31.8	0.160
HDL cholesterol, mg/dL	36.0 (30.0, 41.0)	40.0 (34.7, 46.2)	**0.001**
Triglycerides, mg/dL	147.0 (93.0, 217.0)	130.0 (92.2, 193.0)	0.245
Renal function			
Creatinine, mg/dL	1.0 (0.8, 1.3)	0.9 (0.8, 1.2)	0.051
eGFR, ml/min/1.73m^2^	89.5 (60.0, 102.0)	95.0 (71.8, 104.0)	0.063
UACR, mg/g Cr	25.4 (15.1, 141.0)	23.0 (8.25, 58.0)	0.066
Inflammatory markers			
CRP, mg/dL	0.7 (0.3, 2.4)	0.3 (0.1, 0.6)	**< 0.001**
ESR, mm/h	33.0 (17.0, 64.0)	14.0 (7.0, 35.5)	**< 0.001**
Fibrinogen, mg/dL	431.0 (332.0, 627.0)	349.0 (294.0, 435.0)	**< 0.001**
Blood count			
Red blood cells, 10⁶/uL	4.832 ± 0.786	5.018 ± 0.702	0.203
Hb, g/dL	13.6 (12.3, 14.7)	14.7 (12.8, 15.6)	**< 0.001**
HCT, %	41.5 (37.5, 44.8)	44.0 (40.3, 46.6)	**0.003**
White blood cells, 10³/uL	8.5 (6.93, 10.3)	7.0 (6.0, 8.5)	**< 0.001**
PLT, 10³/uL	239.0 (191.0, 334.0)	222.0 (175.4, 262.5)	**0.042**
Glucose-lowering therapy			
Metformin, n (%)	38 (48.7)	47 (61.0)	0.168
SGLT2-i, n (%)	17 (21.8)	14 (18.2)	0.718
GLP-1RA, n (%)	11 (14.1)	14 (18.2)	0.637
DPP4-i, n (%)	4 (5.12)	2 (2.6)	0.689
Insulin, n (%)	57 (73.1)	48 (62.3)	0.208
Sulfonylureas, n (%)	5 (6.4)	13 (16.9)	0.084
Other therapies			
Diuretic, n (%)	31 (39.7)	25 (32.5)	0.438
β-blockers, n (%)	42 (53.8)	25 (32.5)	**0.012**
ACE-inhibitors, n (%)	22 (28.2)	28 (36.4)	0.360
ARBs, n (%)	33 (42.3)	17 (22.1)	**0.012**
Calcium channel blockers, n (%)	22 (28.2)	17 (22.1)	0.488
Anticoagulant/antiplatelets therapy, n (%)	54 (69.2)	46 (60)	0.286
Lipid-lowering therapy, n (%)	54 (69.2)	44 (57.14)	0.163

Data are reported as mean ± SD, median (IQR) or number and percentage ACE, angiotensin-converting enzyme; ARBs, angiotensin II receptor blockers; BMI, body mass index (calculated as weight in kilograms divided by height in meters squared); CRP, C-reactive protein; DBP, diastolic blood pressure; DPP4-i, dipeptidyl peptidase-4 inhibitors; eGFR, estimated glomerular filtration rate; ESR, erythrocyte sedimentation rate; GLP-1RA, glucagon-like peptide-1 receptor agonist; Hb, haemoglobin; HbA1c, glycated haemoglobin; HCT, haematocrit, HDL-C, high-density lipoprotein cholesterol; LDL-C, low-density lipoprotein cholesterol; PLT, platelets; SBP, systolic blood pressure; SGLT2-i, Sodium-Glucose Cotransporter 2 inhibitors; UACR, urinary albumin to creatinine ratio

**Fig. 1:**
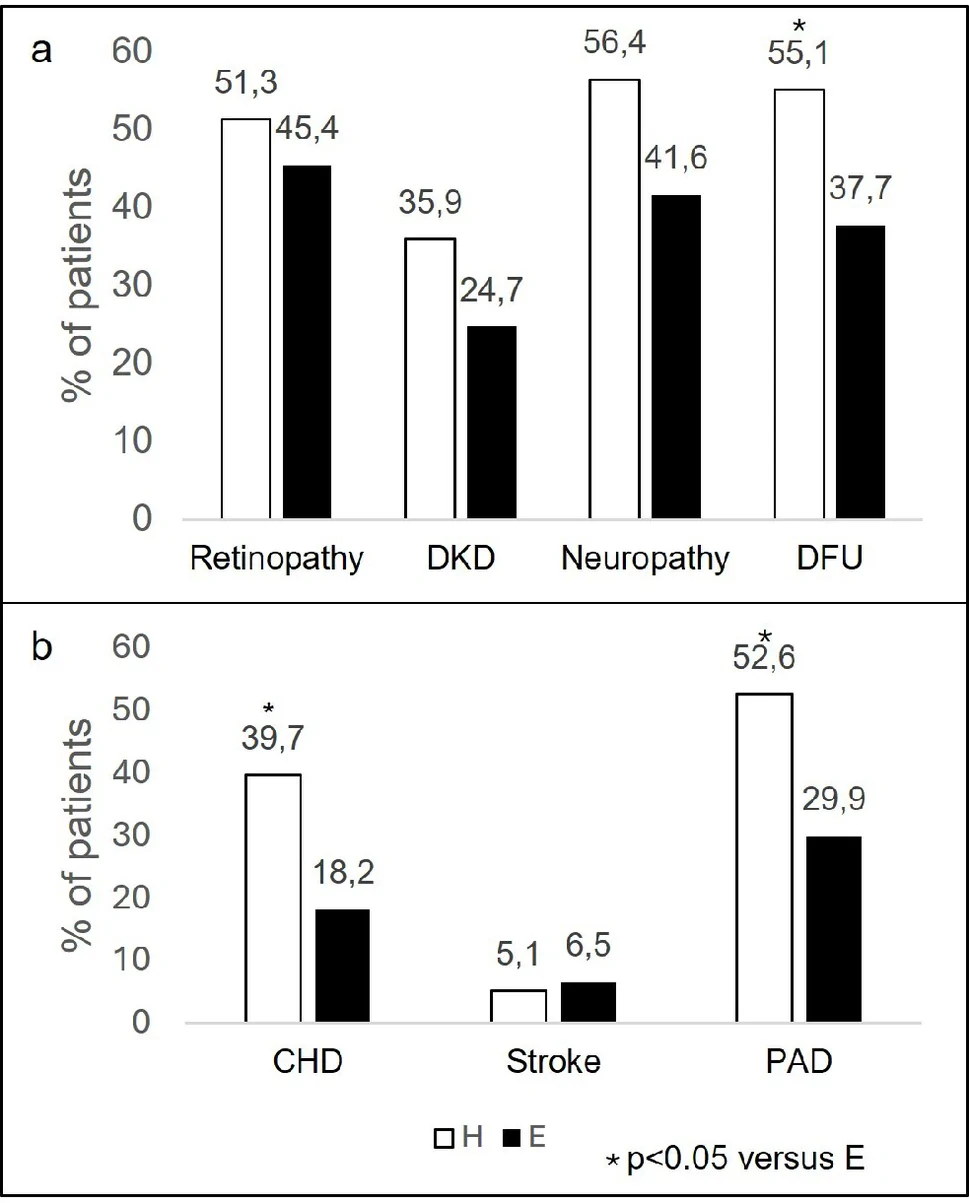
Proportion of diabetic males with microvascular (**a**) and macrovascular (**b**) complications. Black bars indicate eugonadal group (E) and white bars indicate hypogonadal group (H), CHD, coronary heart disease; DFU, diabetic foot ulceration; DKD, diabetic kidney disease; PAD, peripheral artery disease

The proportion of long-term complications is reported in Fig. 1; Table 2. Microvascular complications did not differ between groups, except for a higher proportion of DFU in the H group (*p* = 0.043). With regard to macrovascular complications, the H group presented a significantly higher prevalence of PAD (*p* = 0.007) and coronary heart disease (CHD) (*p* = 0.005). Regarding DFU-related complications, the proportion of patients with osteomyelitis (*p* = 0.045) and those undergoing both minor and major lower-limb amputations was significantly higher in the H group (*p* = 0.004) compared with the E group. No differences between groups were found according to the pathogenesis of DFU (data not shown).

**Table 2:** Clinical characteristics and interventions related to diabetic foot ulcers, according to hypogonadal status

Parameters	Hypogonadal (78)	Eugonadal (77)	*p*-value
Duration of ulcer, months	2.5 (2.0, 5.0)	4.0 (2.25, 9.25)	0.188
Osteomyelitis, n (%)	25 (32.0)	13 (16.9)	**0.045**
Charcot foot, n (%)	5 (6.4)	2 (2.6)	0.450
Amputation, n (%)	14 (18.0)	2 (2.6)	**0.004**
Revascularization, n (%)	8 (10.3)	3 (3.9)	0.219
Debridement/graft, n (%)	19 (24.4)	11 (14.3)	0.166

Data are reported as median (IQR) or number and percentage

Regression analysis is reported in Table 3. Binary logistic regression analysis showed that higher T levels were significantly associated with a reduction in the risk of PAD (*p* = 0.014), CHD (*p* = 0.013), DFU (*p* = 0.007), osteomyelitis (*p* = 0.013) and lower limb amputation (*p* < 0.001). The multiple logistic regression analysis adjusted for all the potential confounding variables showed a significant association between T levels and the risk of both PAD and amputation. The complete step-by-step multiple logistic regression analysis is reported in the Supplementary Materials.

**Table 3:** Multiple logistic regression analysis evaluating the association between T levels and clinically significant outcomes in diabetic men with and without hypogonadism. Odds ratios for testosterone are expressed per 50-unit increase in serum testosterone levels

Binary Logistic Regression			
Variables	OR	95% CI	p-value
PAD	0.905	0.818–0.951	**0.014**
CHD	0.861	0.778–0.951	**0.013**
DFU	0.861	0.778–0.951	**0.007**
Amputation	0.636	0.494–0.818	**< 0.001**
Osteomyelitis	0.861	0.778–0.951	**0.013**
Regression Models Adjusted For HDL-c, Hb, HCT, PLT, age, smoking status, BMI, diabetes duration, HbA1c, eGFR, serum creatinine, UACR, β-blockers, ARBs.			
Variables	OR	95% CI	p-value
PAD	0.861	0.740–0.951	**0.019**
CHD	0.861	0.740–1.051	0.157
DFU	0.861	0.740-1.000	0.051
Amputation	0.575	0.329–0.951	**0.029**
Osteomyelitis	0.951	0.778–1.105	0.405

Odds ratios (OR) refer to the risk change per 50-unit increase in T. Models are adjusted for potential confounding variables: HDL-c, Hb, HCT, PLT, age, smoking status, BMI, diabetes duration, HbA1c, eGFR, serum creatinine, UACR, β-blockers, ARBs. Data are presented as OR with 95% confidence intervals (CI) and p-values. ARBs, angiotensin II receptor blockers; BMI, body mass index; CHD, coronary heart disease; DFU, diabetic foot ulcer; eGFR, estimated glomerular filtration rate; PAD, peripheral artery disease. DFU, diabetic foot ulcer; Hb, haemoglobin; HbA1c, glycosylated haemoglobin, HCT, haematocrit, HDL-C, high-density lipoprotein cholesterol; LDL-C, low-density lipoprotein cholesterol; PAD, peripheral artery disease; PLT, platelets; UACR, urinary albumin-to-creatinine ratio

## Discussion and conclusion

This retrospective, observational cross-sectional and single-centre study showed that LOH was associated with a significant increase of lower-limb complications.

In these individuals, hypogonadism was also associated with changes in blood count, particularly reduction in haemoglobin and haematocrit levels and increase in inflammatory markers, as compared with the eugonadal population. Moreover, low T levels were associated with the presence of PAD and lower-limb amputation, suggesting a potential relationship between hypogonadism and peripheral vascular complications in men with T2D.

These findings are consistent with previous literature showing that T deficiency is associated with increased pro-inflammatory cytokines, whereas T therapy reduces TNF-α, IL-1β, and IL-6 and enhances anti-inflammatory signaling. T has also been proposed to modulate multiple immune and molecular pathways, including cytokine networks involving TNF-α, IL-1β, IL-6, and CRP, thereby attenuating the inflammatory response in chronic inflammatory conditions [28, 29].

It is known that reduction in T levels is often observed in people with poor glycaemic control, and therefore with high HbA1c levels [30]. This may be consistent with the high rate of long-term complications of diabetes in subjects with inadequate glycaemic control, although in our study the groups did not differ in HbA1c values.

Low levels of endogenous T have been directly related to an increased risk of cardiovascular disease in middle-aged and elderly males [31, 32]. Moreover, in men with erectile dysfunction T levels may be independently associated with cardiometabolic conditions, including obesity, diabetes, hypertension, and dyslipidemia, resulting in an increased risk of coronary heart disease [33]. Furthermore, a meta-analysis of 12 community-based studies reported the association with low T levels and both all-cause and cardiovascular mortality [34]. This is relevant since low levels of T and T2D are both risk factors for cardiovascular disease. Although cardiovascular disease is systemic, our findings highlight that the health of lower limb vascular district may be clinically related with the T deficiency. A recent study analysed the relationship between hypogonadism and vascular complications in 261 non-obese subjects with T2D. This study did not show significant differences in microvascular complications, but it reported an increased frequency of macrovascular complications in people with hypogonadism. Moreover, overt hypogonadism, hypertension, hypercholesterolemia, and severe hypoglycaemia were identified as independent risk factors for macrovascular disease [35]. Furthermore, a recent longitudinal study including 625 adult men with a mean age of 65 years from the Baltimore Longitudinal Study of Aging, reported that anaemia, obesity, heart failure, PAD, stroke, and diabetes were inversely associated with total T levels [36]. Our findings align with existing literature, reinforcing the association between T levels, inflammatory markers, and vascular diseases.

To the best of our knowledge, our study is the first to report an association between hypogonadism and lower-limb vascular complications including particularly DFU, PAD, osteomyelitis and amputation. These findings are consistent with previous studies indicating that low testosterone levels are observed in individuals with T2D and are associated with atherosclerotic plaques, endothelial dysfunction, and elevated CRP levels [20, 37].

With regard to PAD, T may exert a long-term influence on arteries, as suggested by evidence supporting that low serum T levels are an independent predictor of the arterial stiffness index [38, 39]. On the other hand, T may induce relaxation of endothelium-denuded coronary arteries through enhanced nitric oxide production [40]. Moreover, physiological concentrations of T may have a positive influence on the haemostatic system modulating anticoagulant activity: particularly, T may stimulate the expression of both tissue factor pathway inhibitor and tissue plasminogen activator (tPA) and inhibit the secretion of plasminogen activator inhibitor type 1 (PAI-1) by human umbilical vein endothelial cells [41, 42]. Furthermore, a cross-sectional observational study involving 64 apparently healthy adult men, demonstrated that central obesity and lower free-T and SHBG levels are associated with increased plasma concentrations of prothrombotic factors, including fibrinogen, factor VII, and PAI-1, placing the study population at high risk of cardiovascular disease [43].

Importantly, in our cohort, the higher inflammatory markers observed in hypogonadal subjects may reflect a complex relationship in which inflammation represents both a shared consequence of metabolic dysfunction and a potential intermediate pathway linking T deficiency to vascular damage. Particularly, chronic vascular disease and systemic inflammation may contribute to suppression of the HPG axis, leading to secondary hypogonadism. On the other hand, hypogonadism may be considered as a marker of adverse metabolic and vascular status.

The present study has limitations: the retrospective, cross-sectional design precludes any inference regarding causality or temporality. Moreover, as a single-centre study including a relatively small cohort, the findings may not be generalisable to the broader population of men with T2D. Furthermore, the study presents a potential selection bias related to the selective inclusion criteria. Indeed, the study population included men with diabetes undergoing hormonal evaluation, either because of symptoms suggestive of endocrine dysfunction or as part of broader clinical assessments in the presence of diabetes-related complications, including vascular disease. This may have contributed to the relatively high prevalence of LOH observed in our cohort. In addition, differences in diagnostic criteria and testosterone thresholds across studies may contribute to variability in the reported prevalence of LOH. The present study adopted the SIAMS/SIE and EAU guideline-recommended thresholds (total testosterone < 230 ng/dL or free testosterone < 220 pmol/L), whereas several prevalence studies have used different cut-offs, potentially influencing prevalence estimates. Finally, LOH-related symptoms were retrospectively derived from clinical records without the use of specific assessment instruments, which may have limited their standardized evaluation. However, to date, few studies have investigated the relationship between T levels and vascular lower limb complications. This is relevant considering the 25% prevalence of hypogonadism in people with T2D [5].

In conclusion, in this cohort of males with T2D, LOH is associated with an unfavourable clinical profile characterized by haematological alterations, increased inflammatory burden, and a higher prevalence of lower-limb vascular complications. In particular, lower T levels were significantly associated with PAD and lower-limb amputation, even after adjustment for relevant confounders. These findings support the role of hypogonadism as a clinical marker of vascular impairment in men with T2D, highlighting the importance of hormonal evaluation in selected high-risk individuals. Further prospective studies are needed to clarify the clinical relevance of LOH in this population.

## Supplementary Information

Below is the link to the electronic supplementary material.

Supplementary Material 1

## Data Availability

Some or all data sets generated during and/or analysed during the present study are not publicly available but are available from the corresponding author on reasonable request.
