# Supplementary material for: Late-onset hypogonadism and lower limb vascular complications in men with type 2 diabetes: an observational study in a tertiary care centre
Source: J Endocrinol Invest. 2026 Jun 26;49(11):3531–9. doi: 10.1007/s40618-026-02967-4 (PMC13722643; doi:10.1007/s40618-026-02967-4)
Supplement: Supplementary file 1 [file 40618_2026_2967_MOESM1_ESM.docx]

**Supplementary Material**

**Table 1.** Multiple logistic regression analysis step-by-step evaluating the association between T levels and clinically significant outcomes in diabetic males with and without hypogonadism.

| **Binary Logistic Regression** | | | |
| --- | --- | --- | --- |
| **Variables** | **OR** | **95% CI** | **p-value** |
| **PAD** | 0.905 | 0.818-0.951 | **0.014** |
| **CHD** | 0.861 | 0.778-0.951 | **0.013** |
| **DFU** | 0.861 | 0.778-0.951 | **0.007** |
| **Amputation** | 0.636 | 0.494-0.818 | **<0.001** |
| **Osteomyelitis** | 0.861 | 0.778-0.951 | **0.013** |
| **Regression Models Adjusted For HDL-c** | | | |
| **Variables** | **OR** | **95% CI** | **p-value** |
| **PAD** | 0.861 | 0.778-0.951 | **0.004** |
| **CHD** | 0.905 | 0.818-1.000 | **0.048** |
| **DFU** | 0.861 | 0.778-0.951 | **0.009** |
| **Amputation** | 0.636 | 0.494-0.861 | **0.001** |
| **Osteomyelitis** | 0.861 | 0.778-1.000 | **0.03** |
| **Regression Models Adjusted For HDL-c, Hb** | | | |
| **Variables** | **OR** | **95% CI** | **p-value** |
| **PAD** | 0.861 | 0.778-1.000 | **0.018** |
| **CHD** | 0.905 | 0.778-1.000 | 0.065 |
| **DFU** | 0.905 | 0.818-1.000 | 0.103 |
| **Amputation** | 0.704 | 0.547-0.905 | **0.005** |
| **Osteomyelitis** | 0.905 | 0.818-1.051 | 0.191 |
| **Regression Models Adjusted For HDL-c, Hb, HCT** | | | |
| **Variables** | **OR** | **95% CI** | **p-value** |
| **PAD** | 0.861 | 0.778-1.000 | **0.018** |
| **CHD** | 0.905 | 0.818-1.000 | 0.079 |
| **DFU** | 0.905 | 0.818-1.000 | 0.109 |
| **Amputation** | 0.704 | 0.547-0.905 | **0.005** |
| **Osteomyelitis** | 0.905 | 0.818-1.051 | 0.185 |
| **Regression Models Adjusted For HDL-c, Hb, HCT, PLT** | | | |
| **Variables** | **OR** | **95% CI** | **p-value** |
| **PAD** | 0.905 | 0.778-1.000 | **0.029** |
| **CHD** | 0.905 | 0.818-1.000 | 0.094 |
| **DFU** | 0.951 | 0.861-1.051 | 0.329 |
| **Amputation** | 0.704 | 0.547-0.951 | **0.016** |
| **Osteomyelitis** | 0.951 | 0.818-1.105 | 0.442 |
| **Regression Models Adjusted For HDL-c, Hb, HCT, PLT, age** | | | |
| **Variables** | **OR** | **95% CI** | **p-value** |
| **PAD** | 0.861 | 0.778-1.000 | **0.023** |
| **CHD** | 0.905 | 0.778-1.000 | 0.097 |
| **DFU** | 0.951 | 0.861-1.051 | 0.329 |
| **Amputation** | 0.74 | 0.547-0.951 | **0.02** |
| **Osteomyelitis** | 0.951 | 0.818-1.105 | 0.419 |
| **Regression Models Adjusted For HDL-c, Hb, HCT, PLT, age, smoking status** | | | |
| **Variables** | **OR** | **95% CI** | **p-value** |
| **PAD** | 0.861 | 0.778-0.951 | **0.016** |
| **CHD** | 0.861 | 0.778-1.000 | 0.055 |
| **DFU** | 0.951 | 0.861-1.051 | 0.38 |
| **Amputation** | 0.704 | 0.547-0.951 | **0.02** |
| **Osteomyelitis** | 0.951 | 0.818-1.105 | 0.449 |
| **Regression Models Adjusted For HDL-c, Hb, HCT, PLT, age, smoking status, BMI** | | | |
| **Variables** | **OR** | **95% CI** | **p-value** |
| **PAD** | 0.861 | 0.740-0.951 | **0.012** |
| **CHD** | 0.861 | 0.740-1.000 | **0.038** |
| **DFU** | 0.905 | 0.818-1.18X10^15^ | 0.204 |
| **Amputation** | 0.704 | 0.520-0.951 | 0.015 |
| **Osteomyelitis** | 0.951 | 0.818-1.051 | 0.37 |
| **Regression Models Adjusted For HDL-c, Hb, HCT, PLT, age, smoking status, BMI, diabetes duration** | | | |
| **Variables** | **OR** | **95% CI** | **p-value** |
| **PAD** | 0.861 | 0.740-0.951 | **0.014** |
| **CHD** | 0.818 | 0.704-0.951 | **0.011** |
| **DFU** | 0.905 | 0.818-1.051 | 0.16 |
| **Amputation** | 0.704 | 0.520-0.951 | **0.032** |
| **Osteomyelitis** | 0.951 | 0.818-1.105 | 0.393 |
| **Regression Models Adjusted For HDL-c, Hb, HCT, PLT, age, smoking status, BMI, diabetes duration, HbA1c** | | | |
| **Variables** | **OR** | **95% CI** | **p-value** |
| **PAD** | 0.861 | 0.740-0.951 | **0.012** |
| **CHD** | 0.818 | 0.740-0.951 | **0.019** |
| **DFU** | 0.861 | 0.778-1.000 | 0.072 |
| **Amputation** | 0.704 | 0.520-0.951 | **0.018** |
| **Osteomyelitis** | 0.905 | 0.778-1.051 | 0.288 |
| **Regression Models Adjusted For HDL-c, Hb, HCT, PLT, age, smoking status, BMI, diabetes duration, HbA1c, eGFR** | | | |
| **Variables** | **OR** | **95% CI** | **p-value** |
| **PAD** | 0.861 | 0.740-0.951 | **0.013** |
| **CHD** | 0.818 | 0.704-0.951 | **0.018** |
| **DFU** | 0.861 | 0.778-1.000 | 0.069 |
| **Amputation** | 0.704 | 0.520-0.951 | **0.02** |
| **Osteomyelitis** | 0.905 | 0.778-1.051 | 0.282 |
| **Regression Models Adjusted For HDL-c, Hb, HCT, PLT, age, smoking status, BMI, diabetes duration, HbA1c, eGFR, creatinine** | | | |
| **Variables** | **OR** | **95% CI** | **p-value** |
| **PAD** | 0.861 | 0.740-0.951 | **0.015** |
| **CHD** | 0.861 | 0.740-1.000 | **0.025** |
| **DFU** | 0.861 | 0.740-1.000 | 0.041 |
| **Amputation** | 0.636 | 0.446-0.905 | **0.01** |
| **Osteomyelitis** | 0.905 | 0.778-1.051 | 0.242 |
| **Regression Models Adjusted For HDL-c, Hb, HCT, PLT, age, smoking status, BMI, diabetes duration, HbA1c, eGFR, creatinine, UACR** | | | |
| **Variables** | **OR** | **95% CI** | **p-value** |
| **PAD** | 0.861 | 0.740-147.270 | **0.017** |
| **CHD** | 0.818 | 0.704-1.000 | **0.029** |
| **DFU** | 0.861 | 0.740-1.000 | 0.079 |
| **Amputation** | 0.547 | 0.329-0.905 | **0.023** |
| **Osteomyelitis** | 0.951 | 0.778-1.105 | 0.293 |
| **Regression Models Adjusted For HDL-c, Hb, HCT, PLT, age, smoking status, BMI, diabetes duration, HbA1c, eGFR, creatinine, UACR, β-blockers** | | | |
| **Variables** | **OR** | **95% CI** | **p-value** |
| **PAD** | 0.861 | 0.740-0.951 | **0.019** |
| **CHD** | 0.861 | 0.740-1.051 | 0.14 |
| **DFU** | 0.861 | 0.740-1.000 | 0.053 |
| **Amputation** | 0.547 | 0.329-0.951 | **0.027** |
| **Osteomyelitis** | 0.951 | 0.778-1.105 | 0.431 |
| **Regression Models Adjusted For HDL-c, Hb, HCT, PLT, age, smoking status, BMI, diabetes duration, HbA1c, eGFR, creatinine, UACR, β-blockers, ARBs** | | | |
| **Variables** | **OR** | **95% CI** | **p-value** |
| **PAD** | 0.861 | 0.740-0.951 | **0.019** |
| **CHD** | 0.861 | 0.740-1.051 | 0.157 |
| **DFU** | 0.861 | 0.740-1.000 | 0.051 |
| **Amputation** | 0.575 | 0.329-0.951 | **0.029** |
| **Osteomyelitis** | 0.951 | 0.778-1.105 | 0.405 |

Odds ratios are expressed per 50-unit increase in serum T levels. Models are progressively adjusted for potential confounding variables. Data are presented as OR with 95% confidence intervals (CI) and p-values. ARBs, angiotensin II receptor blockers; BMI, body mass index; CHD, coronary heart disease; DFU, diabetic foot ulcer; eGFR, estimated glomerular filtration rate; PAD, peripheral artery disease. DFU, diabetic foot ulcer; Hb, haemoglobin; HbA1c, glycosylated haemoglobin, HCT, haematocrit, HDL-C, high-density lipoprotein cholesterol; LDL-C, low-density lipoprotein cholesterol; PAD, peripheral artery disease; PLT, platelets; UACR, urinary albumin-to-creatinine ratio.
